# Characteristics of baseline frequency data in spinal RCTs do not suggest widespread non-random allocation

**DOI:** 10.1007/s00586-023-07813-2

**Published:** 2023-06-12

**Authors:** Manon Malia Sydney Levayer, Gem Rui Ping Chew, Kyle Alexander Sheldrick, Ashish Dhar Diwan

**Affiliations:** 1 School of Biomedical Engineering, The University of Sydney, Darlington, Australia; 2 Faculty of Medicine, University of New South Wales, Kensington, Australia; 3 Spine Labs, University of New South Wales, Suite 16, Kogarah Private Hospital, Kogarah, NSW Australia; 4 Spine Service, St George Hospital Campus, Kogarah, Australia

**Keywords:** Statistical analysis, RCTs, Random allocation, Fraud, Categorical data, Baseline characteristics, Stouffer, Stouffer–Fisher, Systematic review, Spine

## Abstract

**Background:**

Recent signs of fraudulent behaviour in spine RCTs have queried the integrity of trials in the field. RCTs are particularly important due to the weight they are accorded in guiding treatment decisions, and thus, ensuring RCTs’ reliability is crucial. This study investigates the presence of non-random baseline frequency data in purported RCTs published in spine journals.

**Methods:**

A PubMed search was performed to obtain all RCTs published in four spine journals (Spine, The Spine Journal, the Journal of Neurosurgery Spine, and European Spine Journal) between Jan-2016 and Dec-2020. Baseline frequency data were extracted, and variable-wise *p* values were calculated using the Pearson Chi-squared test. These *p* values were combined for each study into study-wise *p* values using the Stouffer method. Studies with *p* values below 0.01 and 0.05 and those above 0.95 and 0.99 were reviewed. Results were compared to Carlisle’s 2017 survey of anaesthesia and critical care medicine RCTs.

**Results:**

One hundred sixty-seven of the 228 studies identified were included. Study-wise *p* values were largely consistent with expected genuine randomized experiments. Slightly more study-wise *p* values above 0.99 were observed than expected, but a number of these had good explanations to account for that excess. The distribution of observed study-wise *p* values was more closely matched to the expected distribution than those in a similar survey of the anaesthesia and critical care medicine literature.

**Conclusion:**

The data surveyed do not show evidence of systemic fraudulent behaviour. Spine RCTs in major spine journals were found to be consistent with genuine random allocation and experimentally derived data.

**Supplementary Information:**

The online version contains supplementary material available at 10.1007/s00586-023-07813-2.

## Introduction

Randomized controlled trials (RCTs) have been an important tool in evaluating medical interventions since they were introduced by Sir Austin Bradford Hill in 1948 [1, 2]. RCTs are based on random allocation to treatment arms which aims to remove confounding and more accurately estimate the effect of interventions [3]. RCTs have significant weight in guiding treatment decisions and as such, factors undermining their reliability are fundamentally damaging to science [4]. Fabricated or manipulated data can distort the evidence base for a treatment, especially where fraudulent data change the results of a meta-analysis [5, 6]. The public is impacted regardless of whether treatment effects are falsely exaggerated or minimized: patients could be denied life-saving or life-changing treatment; or alternately be exposed to danger with no benefit.

A 2022 article by O’Connell et al. [7] identified a set of trials suspected to present fabricated data for the effectiveness of cognitive therapy for back pain. The claimed positive effect for the treatment was markedly superior to any other studies in the field, to the point of possibly changing the conclusions of a meta-analysis. [8] Such academic scandals have seemed few and far between in spine, in particular, compared to other fields such as anaesthesia [9] and more recently, infectious diseases such as COVID-19 [10]. Researchers such as John Carlisle [9, 11] have taken to reviewing trial data integrity in their fields through statistical analyses. These have identified a number of fraudulent trials and have inspired others to do the same for their fields [12, 13].

Carlisle’s method, which is based on the Fisher–Stouffer method [14] (also sometimes referred to as the Stouffer Z-score method) [7], now dubbed the Carlisle–Stouffer method [15], focuses on the extent of the similarity between the trial arms, using patient characteristics at baseline. As arm allocation in RCTs is random, one would expect a uniform distribution of *p* values between 0 and 1, where studies with a *p* value approaching 0 have very dissimilar and studies with a *p* value approaching 1 have very similar groups at baseline and 0.5 neither more similar nor dissimilar than expected. This method uses baseline summary data (either frequency or interval) which is reported in most trials and allows the computation of a ‘study-wise’ *p* value.

The purpose of this research, by applying methods similar to Carlisle’s, is to broadly assess the probity of RCTs being published in spine journals, to ascertain whether the incident noted by O’Connell et al. [7] was isolated or whether baseline characteristic manipulation was common in spine research. It should be noted that this type of analysis cannot accurately distinguish between intentional misconduct and genuine mistakes. It also cannot detect all fraudulent behaviour but attempts to uncover one of the most common signs of fraud, an abnormal baseline similarity between groups. This is a method that was used by O’Connell and team to uncover a major fraud scandal in spine research. This is not to say that a high study-wise *p* value is necessarily proof of fraud. Misconduct must be the conclusion only if no other explanations exist for the results obtained.

We chose to examine frequency data alone at this stage due to some spine journals enforcing a low number of decimal places, or integer reporting only, for interval data; and this would bias the study-wise *p* values towards 1. For example, in a trial with 200 patients per arm, a mean age of 50 and a standard deviation of 10, enforcing mean age reporting as integer-only will result in a p value greater than 0.99 approximately 70% of the time, simply due to the rounding to integer values.

## Methods

### Search strategy, inclusion criteria and data extraction

**Fig. 1:**
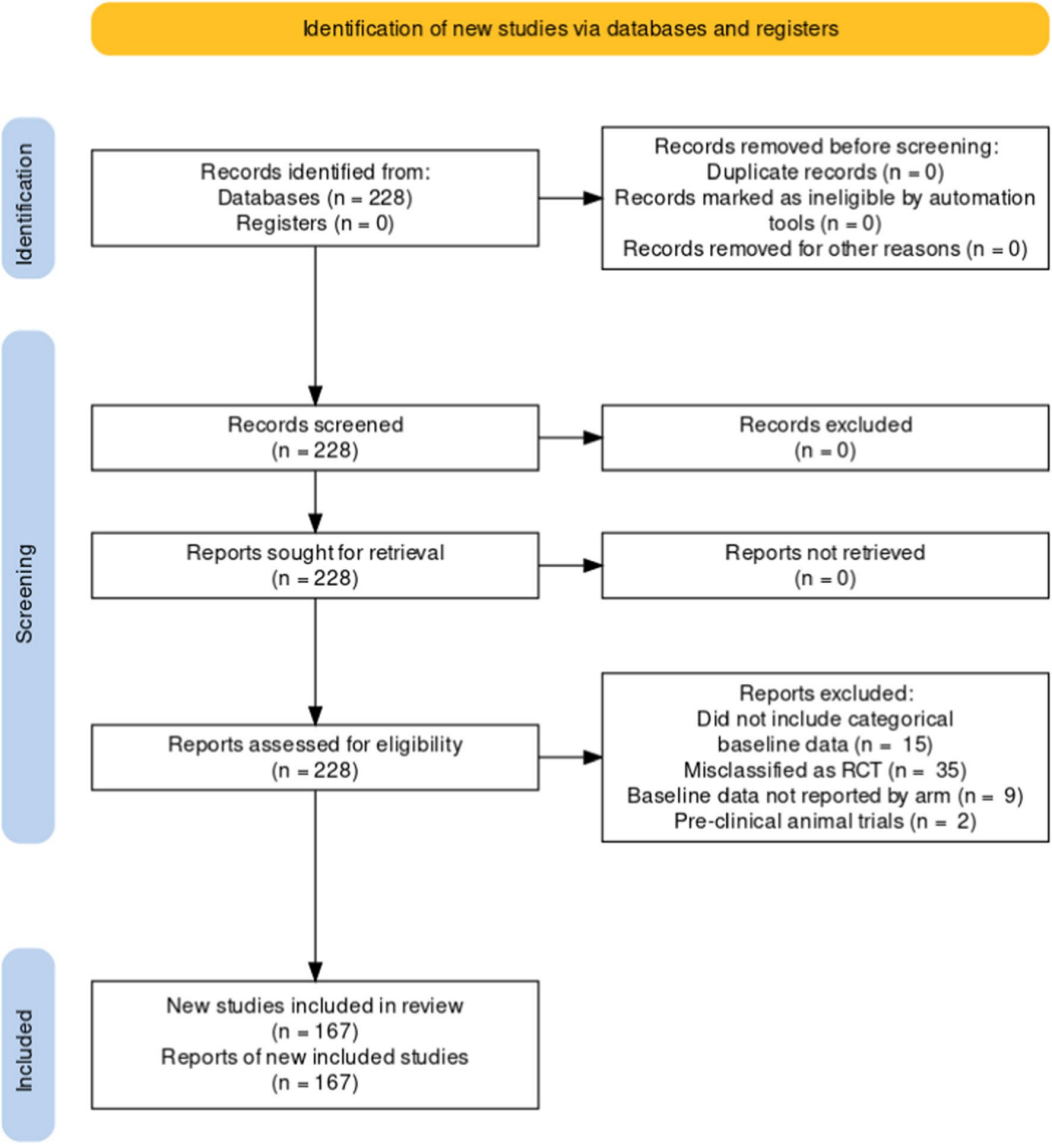
PRISMA flowchart for study inclusion and exclusion. 228 articles were screened from the PubMed search, of which 61 were excluded and 167 included

A PRISMA [16–18] flowchart is presented in Fig. 1. The PubMed electronic database was searched for articles categorized as RCTs in their PubMed metadata published between 2016 and 2020 in four major spine journals (Spine, The Spine Journal, Journal of Neurosurgery Spine, and the European Spine Journal). Each record was reviewed by a single person between June and July 2022 and papers were excluded if the baseline variables were not separately reported by arm. Baseline patient characteristic data were single-extracted from trials which reported at least one baseline frequency variable. A list of all included papers is in Appendix A in supplementary material. Only baseline categorical data (sometimes referred to as frequency variables, where distinct groups are identified, e.g. male/female), recorded before randomization, were included in this analysis.

### Variable-wise p values

The significance of the difference between the arms for each variable was determined using a Pearson Chi-squared test. This test was used regardless of sample size and number of events. While some other tests are preferred in the setting of a small number of events, we prioritized the use of a single test for consistency and comparability. *p* values were calculated for all variables extracted in the previous step. *p* values reported in the articles were not included here.

### Study-wise p values

A Z score was calculated for each *p* value obtained previously, with *p* values of 1 assigned a Z score of 3. For each study, the sum of Z scores was divided by the square root of the number of variables included to obtain a study-wise *p* value. All studies with a *p* value below 0.05 and 0.01, as well as those above 0.95 and 0.99, were noted, and reasons for these *p* values were investigated.

### Statistical analysis

The significance of the difference between the observed and expected number of studies for each p value range was calculated using an exact binomial probability. The distributions of variable-wise and study-wise p values were plotted using Microsoft Excel v16.67.

## Results

### Dataset

**Fig. 2:**
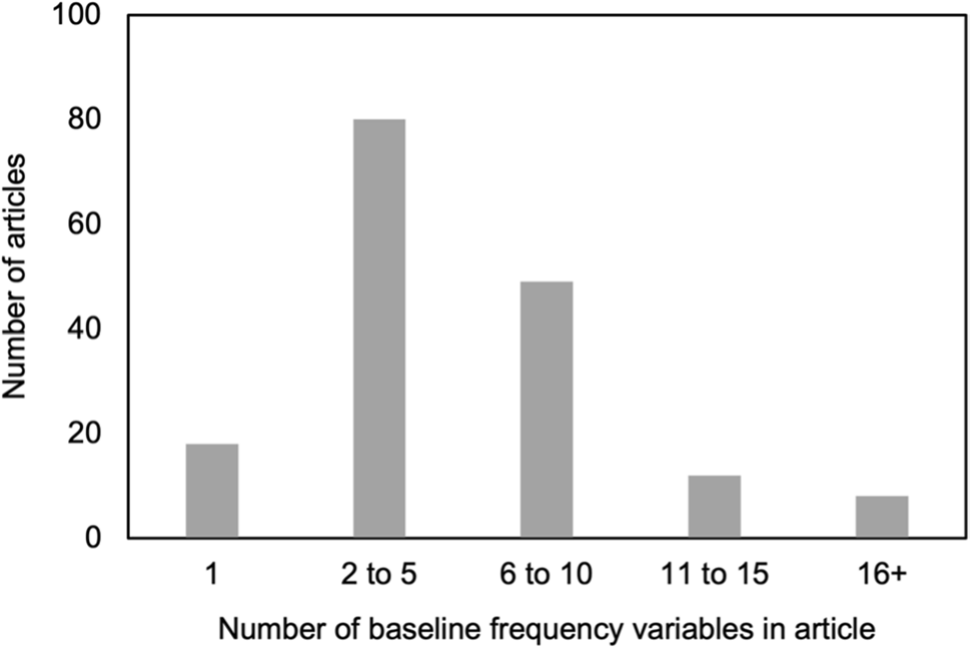
Distribution of the number of baseline frequency variables reported by articles

A total of 228 articles were first identified in the database search (Fig. 1). Sixty-one of those were excluded for reasons including not containing categorical data, not presenting baseline data separately by arm or having been misclassified as an RCT, among others. One hundred sixty-seven articles were retained for analysis, containing a total of 921 categorical variables, for an average 5.5 variables per study. Figure 2 shows the distribution of the number of variables per study.

### Observed p values

**Fig. 3:**
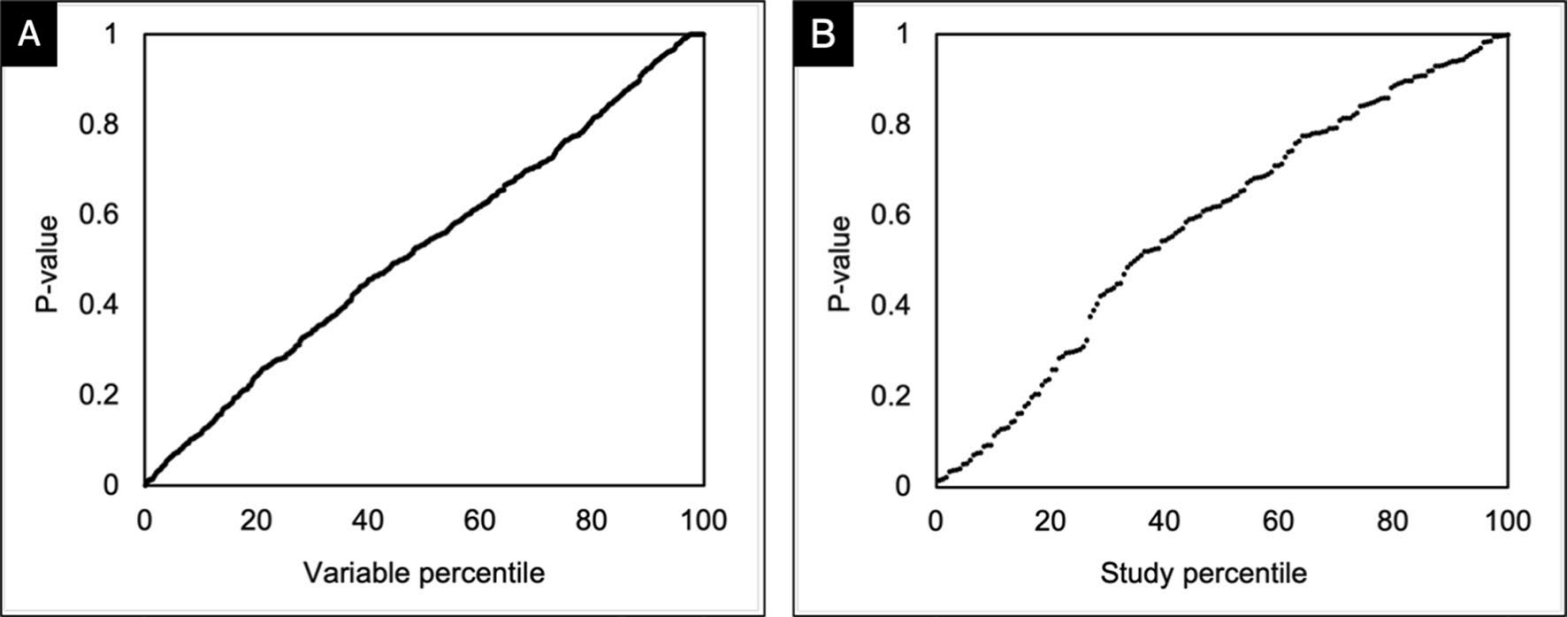
**A** The observed distribution of 921 variable-wise *p* values across 167 trials, plotted by percentile rank. **B** The observed distribution of study-wise *p* values in 167 trials, plotted by percentile rank. The distributions observed are close to the flat distributions expected if all studies report data from genuine randomized experiments. In the setting of systemic fabricated data or non-random allocation, an S shaped curve would be expected

A supplementary file containing all variable-wise and study-wise *p* values is available as Appendix B. Figure 3A and B shows, respectively, all study-wise and variable-wise *p* values, ordered and plotted according to their percentile. Both sets of values show a near-uniform distribution, as would be expected in the setting of genuine random allocation without data fabrication. Only a small tail towards *p* values of 1 (Fig. 3B) was observed. The mean variable-wise *p* values were 0.52, and the mean study-wise *p* value was 0.58.

### Statistical findings

The number of trials with study-wise p values less than 0.01, less than 0.05, and more than 0.95 were not greater than expected by random chance. Only in the *p* > 0.99 category did we observe a significant difference between the observed and expected number of studies: only two were expected, but five were identified (*p* = 0.03) (see Table 1).

**Table 1:** Observed and expected number of studies for each *p* value range

Study-wise p value	*p* < 0.01	*p* < 0.05	0.05 < *p* < 0.95	*p* > 0.95	*p* > 0.99
*n*	0 (0%)	8 (5%)	147 (88%)	12 (7%)	5 (3%)
Expected from binomial distribution	1.67 (1%)	8.35 (5%)	150.3 (90%)	8.35 (5%)	1.67 (1%)
*p* value*	1	0.599	0.837	0.133	0.027**

The group “*p* < 0.05” includes those studies in the group “*p* < 0.01”, likewise the group “*p* > 0.95” includes those studies in the group “*p* > 0.99”

^*^Exact binomial probability of *n* observed events or greater

^**^Statistically significant (*p* < 0.05)

### Comparison to anaesthesia and critical care

**Fig. 4:**
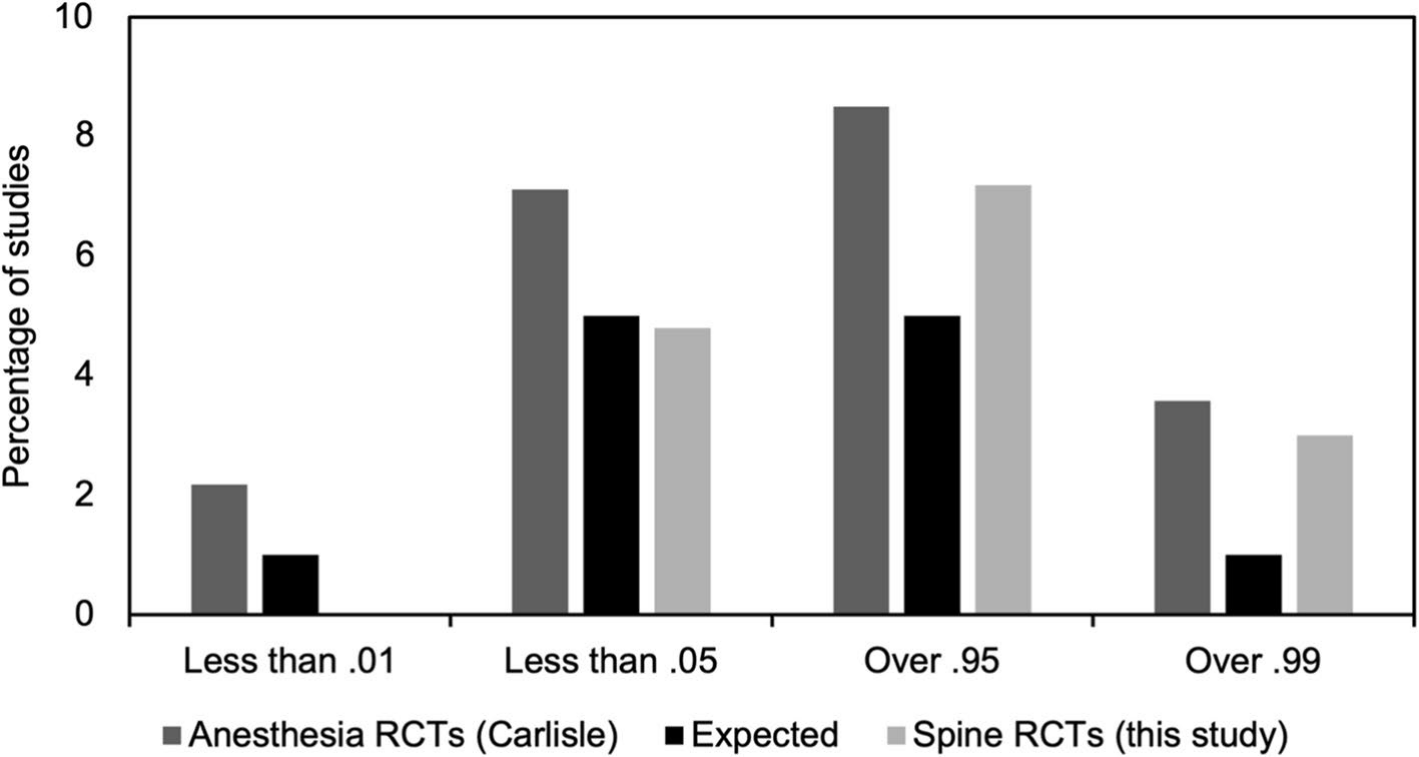
Percentage of *p* values below 0.01 and 0.05 as well as above 0.95 and 0.99 in anaesthesia and critical care RCTs (dark grey), as reported by Carlisle (2017) and spine RCTs (light grey) compared against the expected percentage (black). In all outlying categories, the proportion of spine RCT study-wise p values is similar to or less than that observed in Carlisle

The findings of this study were compared against those of Carlisle’s survey of anaesthesia and critical care in 2017 [11]. In all ‘outlying’ categories, spine presented a lower percentage of RCTs than anaesthesia and critical care (by 2.18%, 2.33%, 1.31% and 0.58% for *p* < 0.01, *p* < 0.05, *p* > 0.95 and *p* > 0.99, respectively) as can be seen in Fig. 4.

## Discussion

Our results are reassuring in that they do not identify evidence of widespread data fabrication or non-random allocation in published randomized controlled trials, at least in the major spine journals examined.

### Explanation for three of the five studies with high p values (p > 0.99)

We did not find more papers with very low p values (suggestive of non-random allocations) than statistically expected (Table 1). A slight preponderance of papers with a *p* value greater than 0.99 (suggesting groups more similar than expected by simple random allocation) was seen. Three of the five studies with a study-wise p value above 0.99 had potential explanations. The first was a CBT trial from the Monticone group, whose work has been thoroughly impugned and was the trigger for initiating this study [7, 19]. Another study had allocated patients to arms using block randomization across different countries, which is equivalent to blocking at a centre level and causes de facto stratification [20]. A third trial had a considerable discontinuity effect from its small number of patients (15 in each arm) [21]. To account for this small population, we conducted a sensitivity analysis by calculating the variable-wise mid-*p* and found a study-wise *p* value of 0.91, which no longer qualifies for the ‘above 0.99’ range. When these three trials are removed from the results, the *p* value for the difference between the observed and expected number of trials with a study-wise *p* value above 0.99 is no longer significant (0.5).

### Why is there less observed fraud in spine research than in other specialties?

The prevalence of ‘abnormal’ *p* values appears to be low in spine RCTs. This is certainly true when compared to anaesthesia (see Fig. 4). It is not possible to provide a definitive explanation for the observed differences between research areas; however, we would speculate about three possible causes. Firstly, there are very few single-surgeon trials in spine research, which may be preventing incidents such as the Fujii scandal [9], where an anaesthetist could claim to see thousands of patients a year, without arousing too much suspicion. Secondly, there may be less pressure on spine clinicians to publish as a tool for career advancement than in some other specialties. Thirdly, many spine conferences and journals are accepting of trials with null or negative findings as a substantive contribution to the literature which may remove some perverse incentives and career risks associated with relying on the inherently uncertain nature of genuine clinical study outcomes.

### Limitations

As mentioned earlier, this analysis is not capable of detecting all fraud and results produced by this study should not be taken as accusations of fraud. Where a trial is genuinely conducted, but outcome data are modified after collection, we would not expect any abnormalities to be detected by this method. This method will detect fraud, provided it presents as non-random allocation, but will not detect weaknesses in study design or interpretation.

Furthermore, our results may not be generalizable. The inclusion criteria limited the analysis to ‘reputable’ journals and the findings presented here may not apply to potentially ‘predatory’ journals, which are associated with a lower quality of research and peer review [7].

## Conclusion

We conclude that overall, baseline data in RCTs published in major spine journals are consistent with genuine random allocation. No evidence of systemic suspicious activity was detected. Readers can thus, while remaining cautious, generally assume that RCTs published in these spine journals are genuine.

## Supplementary Information

Below is the link to the electronic supplementary material.

Supplementary file1 (DOCX 168 kb)

Supplementary file2 (XLSX 436 kb)
