# Supplementary material for: Characteristics of baseline frequency data in spinal RCTs do not suggest widespread non-random allocation
Source: Eur Spine J. 2023 Jun 12:1–6. Online ahead of print. doi: 10.1007/s00586-023-07813-2 (PMC10258745; doi:10.1007/s00586-023-07813-2)
Supplement: Supplementary file 1 — Supplementary file1 (DOCX 168 kb) [file 586_2023_7813_MOESM1_ESM.docx]

**Appendix I**

[1-167]

[1] M. Haas *et al.*, "Dose-response and efficacy of spinal manipulation for care of cervicogenic headache: a dual-center randomized controlled trial," (in eng), *Spine J,* vol. 18, no. 10, pp. 1741-1754, Oct 2018, doi: 10.1016/j.spinee.2018.02.019.

[2] Y. Lindbäck, H. Tropp, P. Enthoven, A. Abbott, and B. Öberg, "PREPARE: presurgery physiotherapy for patients with degenerative lumbar spine disorder: a randomized controlled trial," (in eng), *Spine J,* vol. 18, no. 8, pp. 1347-1355, Aug 2018, doi: 10.1016/j.spinee.2017.12.009.

[3] M. Minetama *et al.*, "Supervised physical therapy vs. home exercise for patients with lumbar spinal stenosis: a randomized controlled trial," *The spine journal,* vol. 19, no. 8, pp. 1310-1318, 2019, doi: 10.1016/j.spinee.2019.04.009.

[4] M. D. Arguisuelas, J. F. Lisón, D. Sánchez-Zuriaga, I. Martínez-Hurtado, and J. Doménech-Fernández, "Effects of Myofascial Release in Nonspecific Chronic Low Back Pain: A Randomized Clinical Trial," (in eng), *Spine (Phila Pa 1976),* vol. 42, no. 9, pp. 627-634, May 1 2017, doi: 10.1097/brs.0000000000001897.

[5] K. Chiba, Y. Matsuyama, T. Seo, and Y. Toyama, "Condoliase for the Treatment of Lumbar Disc Herniation: A Randomized Controlled Trial," (in eng), *Spine (Phila Pa 1976),* vol. 43, no. 15, pp. E869-e876, Aug 1 2018, doi: 10.1097/brs.0000000000002528.

[6] S. Konno, N. Oda, T. Ochiai, and L. Alev, "Randomized, Double-blind, Placebo-controlled Phase III Trial of Duloxetine Monotherapy in Japanese Patients With Chronic Low Back Pain," (in eng), *Spine (Phila Pa 1976),* vol. 41, no. 22, pp. 1709-1717, Nov 15 2016, doi: 10.1097/brs.0000000000001707.

[7] Y. Zheng *et al.*, "Whether Orthotic Management and Exercise are Equally Effective to the Patients With Adolescent Idiopathic Scoliosis in Mainland China?: A Randomized Controlled Trial Study," (in eng), *Spine (Phila Pa 1976),* vol. 43, no. 9, pp. E494-e503, May 1 2018, doi: 10.1097/brs.0000000000002412.

[8] J. G. Khalil *et al.*, "A prospective, randomized, multicenter study of intraosseous basivertebral nerve ablation for the treatment of chronic low back pain," (in eng), *Spine J,* vol. 19, no. 10, pp. 1620-1632, Oct 2019, doi: 10.1016/j.spinee.2019.05.598.

[9] J. Wibault, B. Öberg, Å. Dedering, H. Löfgren, P. Zsigmond, and A. Peolsson, "Structured postoperative physiotherapy in patients with cervical radiculopathy: 6-month outcomes of a randomized clinical trial," (in eng), *J Neurosurg Spine,* vol. 28, no. 1, pp. 1-9, Jan 2018, doi: 10.3171/2017.5.Spine16736.

[10] L. Berglund, B. Aasa, P. Michaelson, and U. Aasa, "Effects of Low-Load Motor Control Exercises and a High-Load Lifting Exercise on Lumbar Multifidus Thickness: A Randomized Controlled Trial," (in eng), *Spine (Phila Pa 1976),* vol. 42, no. 15, pp. E876-e882, Aug 1 2017, doi: 10.1097/brs.0000000000001989.

[11] J. S. Fischgrund *et al.*, "Intraosseous basivertebral nerve ablation for the treatment of chronic low back pain: a prospective randomized double-blind sham-controlled multi-center study," *European Spine Journal,* vol. 27, no. 5, pp. 1146-1156, 2018/05/01 2018, doi: 10.1007/s00586-018-5496-1.

[12] A. M. Castro-Sánchez *et al.*, "Short-term effectiveness of spinal manipulative therapy versus functional technique in patients with chronic nonspecific low back pain: a pragmatic randomized controlled trial," (in eng), *Spine J,* vol. 16, no. 3, pp. 302-12, Mar 2016, doi: 10.1016/j.spinee.2015.08.057.

[13] G. D. Yilmaz Yelvar, Y. Çırak, M. Dalkılınç, Y. Parlak Demir, Z. Guner, and A. Boydak, "Is physiotherapy integrated virtual walking effective on pain, function, and kinesiophobia in patients with non-specific low-back pain? Randomised controlled trial," *European Spine Journal,* vol. 26, no. 2, pp. 538-545, 2017/02/01 2017, doi: 10.1007/s00586-016-4892-7.

[14] J. J. Yue *et al.*, "Five-year Results of a Randomized Controlled Trial for Lumbar Artificial Discs in Single-level Degenerative Disc Disease," *Spine (Philadelphia, Pa. 1976),* vol. 44, no. 24, pp. 1685-1696, 2019, doi: 10.1097/BRS.0000000000003171.

[15] D. Noriega *et al.*, "A prospective, international, randomized, noninferiority study comparing an implantable titanium vertebral augmentation device versus balloon kyphoplasty in the reduction of vertebral compression fractures (SAKOS study)," *The spine journal,* vol. 19, no. 11, pp. 1782-1795, 2019, doi: 10.1016/j.spinee.2019.07.009.

[16] D. C. Raja S, A. P. Shetty, B. Subramanian, R. M. Kanna, and S. Rajasekaran, "A prospective randomized study to analyze the efficacy of balanced pre-emptive analgesia in spine surgery," *The spine journal,* vol. 19, no. 4, pp. 569-577, 2019, doi: 10.1016/j.spinee.2018.10.010.

[17] S.-M. Park *et al.*, "Biportal endoscopic versus microscopic lumbar decompressive laminectomy in patients with spinal stenosis: a randomized controlled trial," *The spine journal,* vol. 20, no. 2, pp. 156-165, 2020, doi: 10.1016/j.spinee.2019.09.015.

[18] C. L. A. Vleggeert-Lankamp *et al.*, "The NECK trial: Effectiveness of anterior cervical discectomy with or without interbody fusion and arthroplasty in the treatment of cervical disc herniation; a double-blinded randomized controlled trial," *The spine journal,* vol. 19, no. 6, pp. 965-975, 2019, doi: 10.1016/j.spinee.2018.12.013.

[19] P. M. Herman, M. L. Anderson, K. J. Sherman, B. H. Balderson, J. A. Turner, and D. C. Cherkin, "Cost-effectiveness of Mindfulness-based Stress Reduction Versus Cognitive Behavioral Therapy or Usual Care Among Adults With Chronic Low Back Pain," *Spine (Philadelphia, Pa. 1976),* vol. 42, no. 20, pp. 1511-1520, 2017, doi: 10.1097/BRS.0000000000002344.

[20] H. Yesil, S. Hepguler, U. Dundar, S. Taravati, and B. Isleten, "Does the Use of Electrotherapies Increase the Effectiveness of Neck Stabilization Exercises for Improving Pain, Disability, Mood, and Quality of life in Chronic Neck Pain? A Randomized, Controlled, Single Blind Study," *Spine (Philadelphia, Pa. 1976),* vol. 43, no. 20, pp. E1174-E1183, 2018, doi: 10.1097/BRS.0000000000002663.

[21] M. Minetama *et al.*, "Therapeutic Advantages of Frequent Physical Therapy Sessions for Patients With Lumbar Spinal Stenosis," *Spine (Philadelphia, Pa. 1976),* vol. 45, no. 11, pp. E639-E646, 2020, doi: 10.1097/BRS.0000000000003363.

[22] L. Berglund, B. Aasa, P. Michaelson, and U. Aasa, "Sagittal lumbo-pelvic alignment in patients with low back pain and the effects of a high-load lifting exercise and individualized low-load motor control exercises: a randomized controlled trial," *The spine journal,* vol. 18, no. 3, p. 399, 2018, doi: 10.1016/j.spinee.2017.07.178.

[23] B. D. C. M. S. Leininger, C. P. T. P. McDonough, R. D. C. M. S. P. Evans, T. S. Tosteson, A. N. A. S. Tosteson, and G. D. C. P. Bronfort, "Cost-effectiveness of spinal manipulative therapy, supervised exercise, and home exercise for older adults with chronic neck pain," *The spine journal,* vol. 16, no. 11, pp. 1292-1304, 2016, doi: 10.1016/j.spinee.2016.06.014.

[24] M. Monticone *et al.*, "Adults with idiopathic scoliosis improve disability after motor and cognitive rehabilitation: results of a randomised controlled trial," *European spine journal,* vol. 25, no. 10, pp. 3120-3129, 2016, doi: 10.1007/s00586-016-4528-y.

[25] Z. Chen *et al.*, "Percutaneous transforaminal endoscopic discectomy compared with microendoscopic discectomy for lumbar disc herniation: 1-year results of an ongoing randomized controlled trial," (in English), *Journal of Neurosurgery: Spine SPI,* vol. 28, no. 3, pp. 300-310, 01 Mar. 2018 2018, doi: 10.3171/2017.7.Spine161434.

[26] R. T. Paulsen, J. Rasmussen, L. Y. Carreon, and M. Ø. Andersen, "Return to work after surgery for lumbar disc herniation, secondary analyses from a randomized controlled trial comparing supervised rehabilitation versus home exercises," *The spine journal,* vol. 20, no. 1, pp. 41-47, 2020, doi: 10.1016/j.spinee.2019.09.019.

[27] A. J. Hahne *et al.*, "Individualized functional restoration as an adjunct to advice for lumbar disc herniation with associated radiculopathy. A preplanned subgroup analysis of a randomized controlled trial," *The spine journal,* vol. 17, no. 3, pp. 346-359, 2017, doi: 10.1016/j.spinee.2016.10.004.

[28] V. M. Nemani, H. J. Kim, C. A. Mina, E. D. Sheha, T. Ross, and O. Boachie-Adjei, "Postoperative Blood Salvage and Autotransfusion for Adult Spinal Deformity: A Randomized Controlled Trial," *Spine (Philadelphia, Pa. 1976),* vol. 45, no. 18, pp. 1247-1252, 2020, doi: 10.1097/BRS.0000000000003176.

[29] Z. Chen *et al.*, "Percutaneous Transforaminal Endoscopic Discectomy Versus Microendoscopic Discectomy for Lumbar Disc Herniation: Two-Year Results of a Randomized Controlled Trial," *Spine (Philadelphia, Pa. 1976),* vol. 45, no. 8, pp. 493-503, 2020, doi: 10.1097/BRS.0000000000003314.

[30] M. Fan *et al.*, "Improved Accuracy of Cervical Spinal Surgery With Robot-Assisted Screw Insertion: A Prospective, Randomized, Controlled Study," *Spine (Philadelphia, Pa. 1976),* vol. 45, no. 5, pp. 285-291, 2020, doi: 10.1097/BRS.0000000000003258.

[31] B. Xu, W.-x. Xu, Y.-j. Lao, W.-g. Ding, D. Lu, and H.-f. Sheng, "Multimodal Nutritional Management in Primary Lumbar Spine Surgery: A Randomized Controlled Trial," *Spine (Philadelphia, Pa. 1976),* vol. 44, no. 14, pp. 967-974, 2019, doi: 10.1097/BRS.0000000000002992.

[32] M. Djurasovic *et al.*, "Randomized trial of Cell Saver in 2- to 3-level lumbar instrumented posterior fusions," *Journal of Neurosurgery: Spine,* vol. 29, no. 5, pp. 582-587, 2018, doi: 10.3171/2018.4.spine18116.

[33] C. Kraiwattanapong, V. Arnuntasupakul, R. Kantawan, P. Woratanarat, G. Keorochana, and N. Langsanam, "Effect of Multimodal Drugs Infiltration on Postoperative Pain in Split Laminectomy of Lumbar Spine: A Randomized Controlled Trial," *Spine (Philadelphia, Pa. 1976),* vol. 45, no. 24, pp. 1687-1695, 2020, doi: 10.1097/BRS.0000000000003679.

[34] M. Farshad, A. Aichmair, F. Wanivenhaus, M. Betz, J. Spirig, and D. E. Bauer, "No benefit of early versus late ambulation after incidental durotomy in lumbar spine surgery: a randomized controlled trial," *European spine journal,* vol. 29, no. 1, pp. 141-146, 2019, doi: 10.1007/s00586-019-06144-5.

[35] A. Peolsson *et al.*, "Postoperative structured rehabilitation in patients undergoing surgery for cervical radiculopathy: a 2-year follow-up of a randomized controlled trial," (in English), *Journal of Neurosurgery: Spine SPI,* vol. 31, no. 1, pp. 60-69, 01 Jul. 2019 2019, doi: 10.3171/2018.12.Spine181258.

[36] A. M. Nene, S. Patil, A. P. Kathare, P. Nagad, A. Nene, and F. Kapadia, "Six versus 12 Months of Anti Tubercular Therapy in Patients With Biopsy Proven Spinal Tuberculosis: A Single Center, Open Labeled, Prospective Randomized Clinical Trial—A Pilot study," *Spine (Philadelphia, Pa. 1976),* vol. 44, no. 1, pp. E1-E6, 2019, doi: 10.1097/BRS.0000000000002811.

[37] C. Zoia, D. Bongetta, C. Alicino, M. Chimenti, R. Pugliese, and P. Gaetani, "Usefulness of corset adoption after single-level lumbar discectomy: a randomized controlled trial," (in English), *Journal of Neurosurgery: Spine SPI,* vol. 28, no. 5, pp. 481-485, 01 May. 2018 2018, doi: 10.3171/2017.8.Spine17370.

[38] W. F. Lavelle, K. D. Riew, A. D. Levi, and J. E. Florman, "Ten-year Outcomes of Cervical Disc Replacement With the BRYAN Cervical Disc: Results From a Prospective, Randomized, Controlled Clinical Trial," *Spine (Philadelphia, Pa. 1976),* vol. 44, no. 9, pp. 601-608, 2019, doi: 10.1097/BRS.0000000000002907.

[39] H. Furunes *et al.*, "Facet Arthropathy Following Disc Replacement Versus Rehabilitation: A Prospective Study With 8-Year Follow-Up," *Spine (Philadelphia, Pa. 1976),* vol. 45, no. 21, pp. 1467-1475, 2020, doi: 10.1097/BRS.0000000000003600.

[40] A. F. A. Allam, T. A. A. Abotakia, and W. Koptan, "Role of Cerebrolysin in cervical spondylotic myelopathy patients: a prospective randomized study," *The spine journal,* vol. 18, no. 7, pp. 1136-1142, 2018, doi: 10.1016/j.spinee.2017.11.002.

[41] Y. Chen *et al.*, "Comparison of Anterior Controllable Antedisplacement and Fusion With Posterior Laminoplasty in the Treatment of Multilevel Cervical Ossification of the Posterior Longitudinal Ligament: A Prospective, Randomized, and Control Study With at Least 1-Year Follow Up," *Spine (Philadelphia, Pa. 1976),* vol. 45, no. 16, pp. 1091-1101, 2020, doi: 10.1097/BRS.0000000000003462.

[42] M. K. Urban *et al.*, "A randomized crossover study of the effects of lidocaine on motor- and sensory-evoked potentials during spinal surgery," *The spine journal,* vol. 17, no. 12, pp. 1889-1896, 2017, doi: 10.1016/j.spinee.2017.06.024.

[43] T. Hasegawa *et al.*, "The Titanium-coated PEEK Cage Maintains Better Bone Fusion With the Endplate Than the PEEK Cage 6 Months After PLIF Surgery: A Multicenter, Prospective, Randomized Study," *Spine (Philadelphia, Pa. 1976),* vol. 45, no. 15, pp. E892-E902, 2020, doi: 10.1097/BRS.0000000000003464.

[44] R. Cecchinato, P. Berjano, A. Zerbi, M. Damilano, A. Redaelli, and C. Lamartina, "Pedicle screw insertion with patient-specific 3D-printed guides based on low-dose CT scan is more accurate than free-hand technique in spine deformity patients: a prospective, randomized clinical trial," *European spine journal,* vol. 28, no. 7, pp. 1712-1723, 2019, doi: 10.1007/s00586-019-05978-3.

[45] C. C. Yu *et al.*, "Intravenous and Oral Tranexamic Acid Are Equivalent at Reducing Blood Loss in Thoracolumbar Spinal Fusion: A Prospective Randomized Trial," *Spine (Philadelphia, Pa. 1976),* vol. 44, no. 11, pp. 755-761, 2019, doi: 10.1097/BRS.0000000000002954.

[46] A. Bharadwaj, G. Khurana, and P. Jindal, "Cervical Spine Movement and Ease of Intubation Using Truview or McCoy Laryngoscope in Difficult Intubation," *Spine (Philadelphia, Pa. 1976),* vol. 41, no. 12, pp. 987-993, 2016, doi: 10.1097/BRS.0000000000001395.

[47] J. Svensson *et al.*, "Neck-Related Headache in Patients With Cervical Disc Disease After Surgery and Physiotherapy: A 1-Year Follow-up of a Prospective Randomized Study," (in eng), *Spine (Phila Pa 1976),* vol. 45, no. 14, pp. 952-959, Jul 15 2020, doi: 10.1097/brs.0000000000003430.

[48] J. Greenwood, A. McGregor, F. Jones, and M. Hurley, "Rehabilitation following lumbar fusion surgery (REFS) a randomised controlled feasibility study," *European spine journal,* vol. 28, no. 4, pp. 735-744, 2019, doi: 10.1007/s00586-019-05913-6.

[49] R. T. Paulsen, L. Y. Carreon, and M. Ø. Andersen, "Patient-reported Outcomes After Surgery for Lumbar Disc Herniation, a Randomized Controlled Trial Comparing the Effects of Referral to Municipal Physical Rehabilitation Versus No Referral," *Spine (Philadelphia, Pa. 1976),* vol. 45, no. 1, pp. 3-9, 2020, doi: 10.1097/BRS.0000000000003221.

[50] C. Pérez-Martínez, K. Gogorza-Arroitaonandia, A. M. Heredia-Rizo, J. Salas-González, and Á. Oliva-Pascual-Vaca, "INYBI: A New Tool for Self-Myofascial Release of the Suboccipital Muscles in Patients With Chronic Non-Specific Neck Pain: A Randomized Controlled Trial," *Spine (Philadelphia, Pa. 1976),* vol. 45, no. 21, pp. E1367-E1375, 2020, doi: 10.1097/BRS.0000000000003605.

[51] J. Li, J.-S. Yang, B.-H. Dong, and J.-M. Ye, "The Effect of Dexmedetomidine Added to Preemptive Ropivacaine Infiltration on Postoperative Pain After Lumbar Fusion Surgery: A Randomized Controlled Trial," *Spine (Philadelphia, Pa. 1976),* vol. 44, no. 19, pp. 1333-1338, 2019, doi: 10.1097/BRS.0000000000003096.

[52] A. B. Jespersen, A. D. K. Andresen, M. K. Jacobsen, M. Ø. Andersen, and L. Y. Carreon, "Does Systemic Administration of Parathyroid Hormone After Noninstrumented Spinal Fusion Surgery Improve Fusion Rates and Fusion Mass in Elderly Patients Compared to Placebo in Patients With Degenerative Lumbar Spondylolisthesis?," *Spine (Philadelphia, Pa. 1976),* vol. 44, no. 3, pp. 157-162, 2019, doi: 10.1097/BRS.0000000000002791.

[53] R. M. D. P. Hedlund, C. M. Johansson, O. M. D. P. Hägg, P. M. D. P. Fritzell, and T. M. D. P. Tullberg, "The long-term outcome of lumbar fusion in the Swedish lumbar spine study," *The spine journal,* vol. 16, no. 5, pp. 579-587, 2015, doi: 10.1016/j.spinee.2015.08.065.

[54] M. Zhang, L. Yan, S. Li, Y. Li, and P. Huang, "Ultrasound-guided transforaminal percutaneous endoscopic lumbar discectomy: a new guidance method that reduces radiation doses," *European Spine Journal,* vol. 28, no. 11, pp. 2543-2550, 2019/11/01 2019, doi: 10.1007/s00586-019-05980-9.

[55] J. N. A. Gibson, A. S. Subramanian, and C. E. H. Scott, "A randomised controlled trial of transforaminal endoscopic discectomy vs microdiscectomy," *European spine journal,* vol. 26, no. 3, pp. 847-856, 2016, doi: 10.1007/s00586-016-4885-6.

[56] R. Nagabhushan, A. P. Shetty, S. R. Dumpa, B. Subarmaniam, R. M. Kanna, and S. Rajasekaran, "Effectiveness and Safety of Batroxobin, Tranexamic acid and a Combination in reduction of blood loss in Lumbar Spinal Fusion Surgery," *Spine (Philadelphia, Pa. 1976),* vol. 43, no. 5, pp. E267-E273, 2017, doi: 10.1097/BRS.0000000000002315.

[57] W.-C. Huang *et al.*, "Effect of Tracheal Intubation Mode on Cuff Pressure During Retractor Splay and Dysphonia Recovery After Anterior Cervical Spine Surgery: A Randomized Clinical Trial," *Spine (Philadelphia, Pa. 1976),* vol. 45, no. 9, pp. 565-572, 2020, doi: 10.1097/BRS.0000000000003339.

[58] M. Takeuchi, N. Wakao, M. Kamiya, A. Hirasawa, K. Murotani, and M. Takayasu, "A double-blind randomized controlled trial of the local application of vancomycin versus ampicillin powder into the operative field for thoracic and/or lumbar fusions," (in English), *Journal of Neurosurgery: Spine SPI,* vol. 29, no. 5, pp. 553-559, 01 Nov. 2018 2018, doi: 10.3171/2018.3.Spine171111.

[59] K. Olmarker, A. Gerward, B. Isberg, A. Lehmann, and S. Berg, "Translational Studies on Biologic Fusion of a Vertebral Segment as a Novel Treatment Modality for Low Back Pain," *Spine (Philadelphia, Pa. 1976),* vol. 45, no. 24, pp. E1636-E1644, 2020, doi: 10.1097/BRS.0000000000003699.

[60] O. K. Jensen, M. H. Andersen, R. D. Østgård, N. T. Andersen, and N. Rolving, "Probiotics for chronic low back pain with type 1 Modic changes: a randomized double-blind, placebo-controlled trial with 1-year follow-up using Lactobacillus Rhamnosis GG," *European spine journal,* vol. 28, no. 11, pp. 2478-2486, 2019, doi: 10.1007/s00586-019-06046-6.

[61] L. S. Aglio *et al.*, "Preemptive analgesia for postoperative pain relief in thoracolumbosacral spine operations: a double-blind, placebo-controlled randomized trial," (in English), *Journal of Neurosurgery: Spine SPI,* vol. 29, no. 6, pp. 647-653, 01 Dec. 2018 2018, doi: 10.3171/2018.5.Spine171380.

[62] S. Ko, S. Chae, W. Choi, and J. Kwon, "Prolonged pain reducing effect of sodium hyaluronate-carboxymethyl cellulose solution in the selective nerve root block (SNRB) of lumbar radiculopathy: a prospective, double-blind, randomized controlled clinical trial," *The spine journal,* vol. 19, no. 4, pp. 578-586, 2019, doi: 10.1016/j.spinee.2018.10.011.

[63] C. Ammendolia *et al.*, "Effect of a prototype lumbar spinal stenosis belt versus a lumbar support on walking capacity in lumbar spinal stenosis: a randomized controlled trial," *The spine journal,* vol. 19, no. 3, pp. 386-394, 2019, doi: 10.1016/j.spinee.2018.07.012.

[64] S. Schmidt, J. Franke, M. Rauschmann, D. Adelt, M. M. Bonsanto, and S. Sola, "Prospective, randomized, multicenter study with 2-year follow-up to compare the performance of decompression with and without interlaminar stabilization," (in eng), *J Neurosurg Spine,* vol. 28, no. 4, pp. 406-415, Apr 2018, doi: 10.3171/2017.11.Spine17643.

[65] B. A. in ’t Veld, T. C. D. Rettig, N. de Heij, J. de Vries, J. F. C. Wolfs, and M. P. Arts, "Maintaining endotracheal tube cuff pressure at 20 mmHg during anterior cervical spine surgery to prevent dysphagia: a double-blind randomized controlled trial," *European spine journal,* vol. 28, no. 2, pp. 353-361, 2018, doi: 10.1007/s00586-018-5798-3.

[66] R. A. Coronado *et al.*, "Early Self-directed Home Exercise Program After Anterior Cervical Discectomy and Fusion: A Pilot Study," *Spine (Philadelphia, Pa. 1976),* vol. 45, no. 4, pp. 217-225, 2020, doi: 10.1097/BRS.0000000000003239.

[67] C. Liao *et al.*, "Modified posterior percutaneous endoscopic cervical discectomy for lateral cervical disc herniation: the vertical anchoring technique," *European spine journal,* vol. 27, no. 6, pp. 1460-1468, 2018, doi: 10.1007/s00586-018-5527-y.

[68] T. Hida *et al.*, "Collar Fixation is not Mandatory after Cervical Laminoplasty: A Randomized Controlled Trial," *Spine (Philadelphia, Pa. 1976),* vol. 42, no. 5, pp. E253-E259, 2016, doi: 10.1097/BRS.0000000000001994.

[69] B. E. Haws *et al.*, "Impact of local steroid application on dysphagia following an anterior cervical discectomy and fusion: results of a prospective, randomized single-blind trial," (in eng), *J Neurosurg Spine,* vol. 29, no. 1, pp. 10-17, Jul 2018, doi: 10.3171/2017.11.Spine17819.

[70] S. He *et al.*, "A Randomized Trial Comparing Clinical Outcomes Between Zero-Profile and Traditional Multilevel Anterior Cervical Discectomy and Fusion Surgery for Cervical Myelopathy," *Spine (Philadelphia, Pa. 1976),* vol. 43, no. 5, pp. E259-E266, 2018, doi: 10.1097/BRS.0000000000002323.

[71] J. Strøm, C. V. Nielsen, L. B. Jørgensen, N. T. Andersen, and M. Laursen, "A web-based platform to accommodate symptoms of anxiety and depression by featuring social interaction and animated information in patients undergoing lumbar spine fusion: a randomized clinical trial," *The spine journal,* vol. 19, no. 5, pp. 827-839, 2019, doi: 10.1016/j.spinee.2018.11.011.

[72] M. Thepsoparn, J. Sereeyotin, and P. Pannangpetch, "Effects of Combined Lower Thoracic Epidural/General Anesthesia on Pain Control in Patients Undergoing Elective Lumbar Spine Surgery: A Randomized Controlled Trial," *Spine (Philadelphia, Pa. 1976),* vol. 43, no. 20, pp. 1381-1385, 2018, doi: 10.1097/BRS.0000000000002662.

[73] G. M. Hardas and G. A. C. Murrell, "A prospective, randomized, double blind, placebo controlled clinical trial assessing the effects of applying a force to C5 by a mechanically assisted instrument (MAI) on referred pain to the shoulder," *Spine (Philadelphia, Pa. 1976),* vol. 43, no. 7, pp. 461-466, 2017, doi: 10.1097/BRS.0000000000002409.

[74] A. V. Gubin, O. G. Prudnikova, K. N. Subramanyam, A. V. Burtsev, M. V. Khomchenkov, and A. V. Mundargi, "Role of closed drain after multi-level posterior spinal surgery in adults: a randomised open-label superiority trial," *European spine journal,* vol. 28, no. 1, pp. 146-154, 2018, doi: 10.1007/s00586-018-5791-x.

[75] H. Furunes *et al.*, "Total disc replacement versus multidisciplinary rehabilitation in patients with chronic low back pain and degenerative discs: 8-year follow-up of a randomized controlled multicenter trial," *The spine journal,* vol. 17, no. 10, pp. 1480-1488, 2017, doi: 10.1016/j.spinee.2017.05.011.

[76] C. Thomé *et al.*, "Annular closure in lumbar microdiscectomy for prevention of reherniation: a randomized clinical trial," *The spine journal,* vol. 18, no. 12, pp. 2278-2287, 2018, doi: 10.1016/j.spinee.2018.05.003.

[77] H. A. G. Soliman, S. Barchi, S. Parent, G. Maurais, A. Jodoin, and J.-M. Mac-Thiong, "Early Impact of Postoperative Bracing on Pain and Quality of Life After Posterior Instrumented Fusion for Lumbar Degenerative Conditions: A Randomized Trial," *Spine (Philadelphia, Pa. 1976),* vol. 43, no. 3, pp. 155-160, 2018, doi: 10.1097/BRS.0000000000002292.

[78] N. Derakhshanrad, H. Saberi, M. S. Yekaninejad, and M. T. Joghataei, "Subcutaneous granulocyte colony-stimulating factor administration for subacute traumatic spinal cord injuries, report of neurological and functional outcomes: a double-blind randomized controlled clinical trial," (in English), *Journal of Neurosurgery: Spine SPI,* vol. 30, no. 1, pp. 19-30, 01 Jan. 2019 2019, doi: 10.3171/2018.6.Spine18209.

[79] B. E. Haws *et al.*, "Impact of local steroid application in a minimally invasive transforaminal lumbar interbody fusion: results of a prospective, randomized, single-blind trial," (in English), *Journal of Neurosurgery: Spine SPI,* vol. 30, no. 2, pp. 222-227, 01 Feb. 2019 2019, doi: 10.3171/2018.7.Spine18584.

[80] J. C. Urquhart *et al.*, "Treatment of thoracolumbar burst fractures: extended follow-up of a randomized clinical trial comparing orthosis versus no orthosis," (in English), *Journal of Neurosurgery: Spine SPI,* vol. 27, no. 1, pp. 42-47, 01 Jul. 2017 2017, doi: 10.3171/2016.11.Spine161031.

[81] A. MacDowall *et al.*, "Artificial disc replacement versus fusion in patients with cervical degenerative disc disease and radiculopathy: a randomized controlled trial with 5-year outcomes," (in eng), *J Neurosurg Spine,* vol. 30, no. 3, pp. 323-331, Jan 11 2019, doi: 10.3171/2018.9.Spine18659.

[82] D. Ovadia, M. Drexler, M. Kramer, A. Herman, and D. E. Lebel, "Closed Wound Subfascial Suction Drainage in Posterior Fusion Surgery for Adolescent Idiopathic Scoliosis: A Prospective Randomized Control Study," *Spine (Philadelphia, Pa. 1976),* vol. 44, no. 6, pp. 377-383, 2019, doi: 10.1097/BRS.0000000000002892.

[83] S. Ruatti *et al.*, "Interest of intra-operative 3D imaging in spine surgery: a prospective randomized study," *European spine journal,* vol. 25, no. 6, pp. 1738-1744, 2015, doi: 10.1007/s00586-015-4141-5.

[84] S.-W. Feng, M.-C. Chang, P.-H. Chou, H.-H. Lin, S.-T. Wang, and C.-L. Liu, "Implantation of an empty polyetheretherketone cage in anterior cervical discectomy and fusion: a prospective randomised controlled study with 2 years follow-up," *European spine journal,* vol. 27, no. 6, pp. 1358-1364, 2018, doi: 10.1007/s00586-017-5450-7.

[85] D. G. Lee, S. H. Ahn, Y. W. Cho, K. H. Do, S. G. Kwak, and M. C. Chang, "Comparison of Intra-articular Thoracic Facet Joint Steroid Injection and Thoracic Medial Branch Block for the Management of Thoracic Facet Joint Pain," *Spine (Philadelphia, Pa. 1976),* vol. 43, no. 2, pp. 76-80, 2018, doi: 10.1097/BRS.0000000000002269.

[86] T. H. Lanman, J. K. Burkus, R. G. Dryer, M. F. Gornet, J. McConnell, and S. D. Hodges, "Long-term clinical and radiographic outcomes of the Prestige LP artificial cervical disc replacement at 2 levels: results from a prospective randomized controlled clinical trial," (in English), *Journal of Neurosurgery: Spine SPI,* vol. 27, no. 1, pp. 7-19, 01 Jul. 2017 2017, doi: 10.3171/2016.11.Spine16746.

[87] J. P. Y. Cheung, D. Samartzis, K. Yeung, M. To, K. D. K. Luk, and K. M.-C. Cheung, "A randomized double-blinded clinical trial to evaluate the safety and efficacy of a novel superelastic nickel–titanium spinal rod in adolescent idiopathic scoliosis: 5-year follow-up," *European spine journal,* vol. 27, no. 2, pp. 327-339, 2017, doi: 10.1007/s00586-017-5245-x.

[88] N. Derakhshanrad, H. Saberi, M. S. Yekaninejad, M. T. Joghataei, and A. Sheikhrezaei, "Granulocyte-colony stimulating factor administration for neurological improvement in patients with postrehabilitation chronic incomplete traumatic spinal cord injuries: a double-blind randomized controlled clinical trial," (in English), *Journal of Neurosurgery: Spine SPI,* vol. 29, no. 1, pp. 97-107, 01 Jul. 2018 2018, doi: 10.3171/2017.11.Spine17769.

[89] C. Y. W. Chan *et al.*, "The Usage of Chewing Gum in Posterior Spinal Fusion Surgery for Adolescent Idiopathic Scoliosis: A Randomized Controlled Trial," *Spine (Philadelphia, Pa. 1976),* vol. 42, no. 19, pp. 1427-1433, 2017, doi: 10.1097/BRS.0000000000002135.

[90] S.-J. Hyun, K.-J. Kim, T.-A. Jahng, and H.-J. Kim, "Minimally Invasive Robotic Versus Open Fluoroscopic-guided Spinal Instrumented Fusions: A Randomized Controlled Trial," *Spine (Philadelphia, Pa. 1976),* vol. 42, no. 6, pp. 353-358, 2017, doi: 10.1097/BRS.0000000000001778.

[91] M. Scholz, F. Kandziora, T. Tschauder, M. Kremer, and A. Pingel, "Prospective randomized controlled comparison of posterior vs. posterior–anterior stabilization of thoracolumbar incomplete cranial burst fractures in neurological intact patients: the RASPUTHINE pilot study," (in English), *European Spine Journal,* vol. 27, no. 12, pp. 3016-3024, Dec 2018

2018-11-29 2018, doi: <https://doi.org/10.1007/s00586-017-5356-4>.

[92] K.-T. Kim *et al.*, "The effectiveness of low-dose and high-dose tranexamic acid in posterior lumbar interbody fusion: a double-blinded, placebo-controlled randomized study," *European spine journal,* vol. 26, no. 11, pp. 2851-2857, 2017, doi: 10.1007/s00586-017-5230-4.

[93] A. J. Hahne *et al.*, "Who Benefits Most From Individualized Physiotherapy or Advice for Low Back Disorders? A Preplanned Effect Modifier Analysis of a Randomized Controlled Trial," *Spine (Philadelphia, Pa. 1976),* vol. 42, no. 21, pp. E1215-E1224, 2017, doi: 10.1097/BRS.0000000000002148.

[94] R. D. Guyer *et al.*, "Five-Year Follow-Up of a Prospective, Randomized Trial Comparing Two Lumbar Total Disc Replacements," *Spine (Philadelphia, Pa. 1976),* vol. 41, no. 1, pp. 3-8, 2016, doi: 10.1097/BRS.0000000000001168.

[95] S. M. Shin, S. G. Kwak, D. G. Lee, and M. C. Chang, "Clinical Effectiveness of Intra-articular Pulsed Radiofrequency Compared to Intra-articular Corticosteroid Injection for Management of Atlanto-occipital Joint Pain: A Prospective Randomized Controlled Pilot Study," *Spine (Philadelphia, Pa. 1976),* vol. 43, no. 11, pp. 741-746, 2018, doi: 10.1097/BRS.0000000000002414.

[96] H. Balling, "Additional Sacroplasty Does Not Improve Clinical Outcome in Minimally Invasive Navigation-Assisted Screw Fixation Procedures for Nondisplaced Insufficiency Fractures of the Sacrum," *Spine (Philadelphia, Pa. 1976),* vol. 44, no. 8, pp. 534-542, 2019, doi: 10.1097/BRS.0000000000002899.

[97] X. Bonfill *et al.*, "Efficacy and safety of urinary catheters with silver alloy coating in patients with spinal cord injury: a multicentric pragmatic randomized controlled trial. The ESCALE trial," *The spine journal,* vol. 17, no. 11, pp. 1650-1657, 2017, doi: 10.1016/j.spinee.2017.05.025.

[98] M. P. Arts, J. F. C. Wolfs, and T. P. Corbin, "Porous silicon nitride spacers versus PEEK cages for anterior cervical discectomy and fusion: clinical and radiological results of a single-blinded randomized controlled trial," *European spine journal,* vol. 26, no. 9, pp. 2372-2379, 2017, doi: 10.1007/s00586-017-5079-6.

[99] M. Coughlan *et al.*, "A Prospective, Randomized, Multicenter Study Comparing Silicated Calcium Phosphate versus BMP-2 Synthetic Bone Graft in Posterolateral Instrumented Lumbar Fusion for Degenerative Spinal Disorders," *Spine (Philadelphia, Pa. 1976),* vol. 43, no. 15, pp. E860-E868, 2018, doi: 10.1097/BRS.0000000000002678.

[100] P. K. Pandey, I. Pawar, J. Gupta, and R. R. Verma, "Comparison of Outcomes of Single-Level Anterior Cervical Discectomy With Fusion and Single-Level Artificial Cervical Disc Replacement for Single-Level Cervical Degenerative Disc Disease," *Spine (Philadelphia, Pa. 1976),* vol. 42, no. 1, pp. E41-E49, 2017, doi: 10.1097/BRS.0000000000001696.

[101] D. Rhon, R. Miller, and J. Fritz, "Effectiveness and Downstream Healthcare Utilization for Patients that Received Early Physical Therapy Versus Usual Care for Low Back Pain: A Randomized Clinical Trial," *Spine (Philadelphia, Pa. 1976),* vol. 43, no. 19, pp. 1313-1321, 2018, doi: 10.1097/BRS.0000000000002619.

[102] H.-J. Kim, H. S. Ahn, Y. Nam, B.-S. Chang, C.-K. Lee, and J. S. Yeom, "Comparative study of the efficacy of transdermal buprenorphine patches and prolonged-release tramadol tablets for postoperative pain control after spinal fusion surgery: a prospective, randomized controlled non-inferiority trial," *European spine journal,* vol. 26, no. 11, pp. 2961-2968, 2017, doi: 10.1007/s00586-017-5213-5.

[103] B. Xu *et al.*, "Continuous wound infusion of ropivacaine for the control of pain after thoracolumbar spinal surgery: a randomized clinical trial," *European spine journal,* vol. 26, no. 3, pp. 825-831, 2015, doi: 10.1007/s00586-015-3979-x.

[104] S. P. Uthaikhup, J. M. Assapun, K. M. D. Watcharasaksilp, and G. P. Jull, "Effectiveness of physiotherapy for seniors with recurrent headaches associated with neck pain and dysfunction: a randomized controlled trial," *The spine journal,* vol. 17, no. 1, pp. 46-55, 2016, doi: 10.1016/j.spinee.2016.08.008.

[105] J. H. Cho *et al.*, "Efficacy of Escherichia coli-derived recombinant human bone morphogenetic protein-2 in posterolateral lumbar fusion: an open, active-controlled, randomized, multicenter trial," *The spine journal,* vol. 17, no. 12, pp. 1866-1874, 2017, doi: 10.1016/j.spinee.2017.06.023.

[106] M. F. Gornet *et al.*, "Cervical disc arthroplasty with the Prestige LP disc versus anterior cervical discectomy and fusion, at 2 levels: results of a prospective, multicenter randomized controlled clinical trial at 24 months," (in eng), *J Neurosurg Spine,* vol. 26, no. 6, pp. 653-667, Jun 2017, doi: 10.3171/2016.10.Spine16264.

[107] L. Helenius, A. Puhakka, T. Manner, O. Pajulo, and I. Helenius, "Preoperative pregabalin has no effect on intraoperative neurophysiological monitoring in adolescents undergoing posterior spinal fusion for spinal deformities: a double-blind, randomized, placebo-controlled clinical trial," *European spine journal,* vol. 27, no. 2, pp. 298-304, 2017, doi: 10.1007/s00586-017-5396-9.

[108] J. Sundseth *et al.*, "The Norwegian Cervical Arthroplasty Trial (NORCAT): 2-year clinical outcome after single-level cervical arthroplasty versus fusion—a prospective, single-blinded, randomized, controlled multicenter study," *European spine journal,* vol. 26, no. 4, pp. 1225-1235, 2016, doi: 10.1007/s00586-016-4922-5.

[109] G. Kubota *et al.*, "Platelet-rich plasma enhances bone union in posterolateral lumbar fusion: A prospective randomized controlled trial," *The spine journal,* vol. 19, no. 2, pp. e34-e40, 2019, doi: 10.1016/j.spinee.2017.07.167.

[110] V. Challier *et al.*, "One-Level Lumbar Degenerative Spondylolisthesis and Posterior Approach: Is Transforaminal Lateral Interbody Fusion Mandatory?: A Randomized Controlled Trial With 2-Year Follow-Up," *Spine (Philadelphia, Pa. 1976),* vol. 42, no. 8, pp. 531-539, 2017, doi: 10.1097/BRS.0000000000001857.

[111] B.-G. Suh, M.-W. Ahn, H.-J. Kim, J. S. Yeom, and G. W. Lee, "Wedge-shaped Resection of the Posterior Bony Arch during Open Door Laminoplasty to Prevent Postoperative Motion Limitation," *Spine (Philadelphia, Pa. 1976),* vol. 42, no. 3, pp. 143-150, 2016, doi: 10.1097/BRS.0000000000001689.

[112] K. Radcliff, D. Coric, and T. Albert, "Five-year clinical results of cervical total disc replacement compared with anterior discectomy and fusion for treatment of 2-level symptomatic degenerative disc disease: a prospective, randomized, controlled, multicenter investigational device exemption clinical trial," (in eng), *J Neurosurg Spine,* vol. 25, no. 2, pp. 213-24, Aug 2016, doi: 10.3171/2015.12.Spine15824.

[113] B. Sturesson, D. Kools, R. Pflugmacher, A. Gasbarrini, D. Prestamburgo, and J. Dengler, "Six-month outcomes from a randomized controlled trial of minimally invasive SI joint fusion with triangular titanium implants vs conservative management," *European spine journal,* vol. 26, no. 3, pp. 708-719, 2016, doi: 10.1007/s00586-016-4599-9.

[114] H.-J. M. D. Kim *et al.*, "Comparative study of the efficacy of limaprost and pregabalin as single agents and in combination for the treatment of lumbar spinal stenosis: a prospective, double blind, randomized controlled non-inferiority trial," *The spine journal,* vol. 16, no. 6, pp. 756-763, 2016, doi: 10.1016/j.spinee.2016.02.049.

[115] O. Ilves *et al.*, "Quality of life and disability: can they be improved by active postoperative rehabilitation after spinal fusion surgery in patients with spondylolisthesis? A randomised controlled trial with 12-month follow-up," *European spine journal,* vol. 26, no. 3, pp. 777-784, 2017, doi: 10.1007/s00586-016-4789-5.

[116] T. Y. Wang *et al.*, "Internally Randomized Control Trial of Radiation Exposure Using Ultra-low Radiation Imaging Versus Traditional C-arm Fluoroscopy for Patients Undergoing Single-level Minimally Invasive Transforaminal Lumbar Interbody Fusion," *Spine (Philadelphia, Pa. 1976),* vol. 42, no. 4, pp. 217-223, 2017, doi: 10.1097/BRS.0000000000001720.

[117] F. Krappel *et al.*, "Herniectomy versus herniectomy with the DIAM spinal stabilization system in patients with sciatica and concomitant low back pain: results of a prospective randomized controlled multicenter trial," *European spine journal,* vol. 26, no. 3, pp. 865-876, 2016, doi: 10.1007/s00586-016-4796-6.

[118] M. Engquist *et al.*, "A 5- to 8-year randomized study on the treatment of cervical radiculopathy: anterior cervical decompression and fusion plus physiotherapy versus physiotherapy alone," (in eng), *J Neurosurg Spine,* vol. 26, no. 1, pp. 19-27, Jan 2017, doi: 10.3171/2016.6.Spine151427.

[119] N. Pireau, V. Cordemans, X. Banse, N. Irda, S. Lichtherte, and L. Kaminski, "Radiation dose reduction in thoracic and lumbar spine instrumentation using navigation based on an intraoperative cone beam CT imaging system: a prospective randomized clinical trial," *European spine journal,* vol. 26, no. 11, pp. 2818-2827, 2017, doi: 10.1007/s00586-017-5229-x.

[120] H. Furunes *et al.*, "Adjacent Disc Degeneration After Lumbar Total Disc Replacement or Non-operative Treatment: A Randomized Study With Eight-year Follow-up," *Spine (Philadelphia, Pa. 1976),* 2018, doi: 10.1097/BRS.0000000000002712.

[121] R. J. Jackson *et al.*, "Subsequent surgery rates after cervical total disc replacement using a Mobi-C Cervical Disc Prosthesis versus anterior cervical discectomy and fusion: a prospective randomized clinical trial with 5-year follow-up," (in eng), *J Neurosurg Spine,* vol. 24, no. 5, pp. 734-45, May 2016, doi: 10.3171/2015.8.Spine15219.

[122] Y.-q. Jiang *et al.*, "A prospective randomized trial comparing anterior cervical discectomy and fusion versus plate-only open-door laminoplasty for the treatment of spinal stenosis in degenerative diseases," *European spine journal,* vol. 26, no. 4, pp. 1162-1172, 2016, doi: 10.1007/s00586-016-4878-5.

[123] M. R. Farrokhi, M. Lotfi, M. S. Masoudi, and M. Gholami, "Effects of methylene blue on postoperative low-back pain and functional outcomes after lumbar open discectomy: a triple-blind, randomized placebo-controlled trial," (in eng), *J Neurosurg Spine,* vol. 24, no. 1, pp. 7-15, Jan 2016, doi: 10.3171/2015.3.Spine141172.

[124] J. M. van Dongen *et al.*, "Cost-effectiveness of manual therapy versus physiotherapy in patients with sub-acute and chronic neck pain: a randomised controlled trial," *European spine journal,* vol. 25, no. 7, pp. 2087-2096, 2016, doi: 10.1007/s00586-016-4526-0.

[125] C. C. Edwards, C. Dean, C. C. Edwards, D. Phillips, and A. Blight, "Can Dysphagia Following Anterior Cervical Fusions With rhBMP-2 Be Reduced With Local Depomedrol Application?: A Prospective, Randomized, Placebo-Controlled, Double-Blind Trial," *Spine (Philadelphia, Pa. 1976),* vol. 41, no. 7, pp. 555-562, 2016, doi: 10.1097/BRS.0000000000001284.

[126] K. Okmen and B. M. Okmen, "The efficacy of interlaminar epidural steroid administration in multilevel intervertebral disc disease with chronic low back pain; a randomized, blinded, prospective study," *The spine journal,* vol. 17, no. 2, pp. 168-174, 2016, doi: 10.1016/j.spinee.2016.08.024.

[127] J. Shi, X. Yue, N. Niu, C. Zhao, H. Qiu, and Z. Wang, "Application of a modified thoracoabdominal approach that avoids cutting open the costal portion of diaphragm during anterior thoracolumbar spine surgery," *European spine journal,* vol. 26, no. 7, pp. 1852-1861, 2016, doi: 10.1007/s00586-016-4917-2.

[128] H. S. Yanik, I. E. Ketenci, T. Coskun, A. Ulusoy, and S. Erdem, "Selection of distal fusion level in posterior instrumentation and fusion of Scheuermann kyphosis: is fusion to sagittal stable vertebra necessary?," *European spine journal,* vol. 25, no. 2, pp. 583-589, 2015, doi: 10.1007/s00586-015-4123-7.

[129] T. P. Loumeau *et al.*, "A RCT comparing 7-year clinical outcomes of one level symptomatic cervical disc disease (SCDD) following ProDisc-C total disc arthroplasty (TDA) versus anterior cervical discectomy and fusion (ACDF)," *European spine journal,* vol. 25, no. 7, pp. 2263-2270, 2016, doi: 10.1007/s00586-016-4431-6.

[130] R. E. Isaacs, J. N. Sembrano, and A. G. Tohmeh, "Two-Year Comparative Outcomes of MIS Lateral and MIS Transforaminal Interbody Fusion in the Treatment of Degenerative Spondylolisthesis: Part II: Radiographic Findings," *Spine (Philadelphia, Pa. 1976),* vol. 41 Suppl 8, pp. S133-S144, 2016, doi: 10.1097/BRS.0000000000001472.

[131] E. L. Werner, K. Storheim, I. Løchting, T. Wisløff, and M. Grotle, "Cognitive Patient Education for Low Back Pain in Primary Care: A Cluster Randomized Controlled Trial and Cost-Effectiveness Analysis," *Spine (Philadelphia, Pa. 1976),* vol. 41, no. 6, pp. 455-462, 2016, doi: 10.1097/BRS.0000000000001268.

[132] B. Fordham, C. Ji, Z. Hansen, R. Lall, and S. E. Lamb, "Explaining How Cognitive Behavioral Approaches Work for Low Back Pain: Mediation Analysis of the Back Skills Training Trial," *Spine (Philadelphia, Pa. 1976),* vol. 42, no. 17, pp. E1031-E1039, 2017, doi: 10.1097/BRS.0000000000002066.

[133] A. T. Al-Shareef, M. T. A. Omar, and A. H. M. Ibrahim, "Effect of kinesio taping on pain and functional disability in chronic nonspecific low back pain," *Spine (Philadelphia, Pa. 1976),* vol. 41, no. 14, pp. E821-E828, 2016, doi: 10.1097/BRS.0000000000001447.

[134] B. Wang, H. Guo, L. Yuan, D. Huang, H. Zhang, and D. Hao, "A prospective randomized controlled study comparing the pain relief in patients with osteoporotic vertebral compression fractures with the use of vertebroplasty or facet blocking," *European spine journal,* vol. 25, no. 11, pp. 3486-3494, 2016, doi: 10.1007/s00586-016-4425-4.

[135] P. M. Arnold *et al.*, "Efficacy of i-Factor Bone Graft versus Autograft in Anterior Cervical Discectomy and Fusion: Results of the Prospective, Randomized, Single-blinded Food and Drug Administration Investigational Device Exemption Study," *Spine (Philadelphia, Pa. 1976),* vol. 41, no. 13, pp. 1075-1083, 2016, doi: 10.1097/BRS.0000000000001466.

[136] A. El Barzouhi *et al.*, "Prognostic value of magnetic resonance imaging findings in patients with sciatica," (in eng), *J Neurosurg Spine,* vol. 24, no. 6, pp. 978-85, Jun 2016, doi: 10.3171/2015.10.Spine15858.

[137] S. E. Reme *et al.*, "Cognitive Interventions and Nutritional Supplements (The CINS Trial): A Randomized Controlled, Multicenter Trial Comparing a Brief Intervention With Additional Cognitive Behavioral Therapy, Seal Oil, and Soy Oil for Sick-Listed Low Back Pain Patients," *Spine (Philadelphia, Pa. 1976),* vol. 41, no. 20, pp. 1557-1564, 2016, doi: 10.1097/BRS.0000000000001596.

[138] D. Fukui *et al.*, "Reduced blood loss and operation time in lumbar posterolateral fusion using a bipolar sealer," *European spine journal,* vol. 26, no. 3, pp. 726-732, 2016, doi: 10.1007/s00586-016-4636-8.

[139] C.-W. C. Lin *et al.*, "The economic burden of guideline-recommended first line care for acute low back pain," *European spine journal,* vol. 27, no. 1, pp. 109-116, 2016, doi: 10.1007/s00586-016-4781-0.

[140] J. Glaser *et al.*, "Motor/Prefrontal Transcranial Direct Current Stimulation (tDCS) Following Lumbar Surgery Reduces Postoperative Analgesia Use," *Spine (Philadelphia, Pa. 1976),* vol. 41, no. 10, pp. 835-839, 2016, doi: 10.1097/BRS.0000000000001525.

[141] N. Rolving, R. Sogaard, C. V. Nielsen, F. B. Christensen, C. Bünger, and L. G. Oestergaard, "Preoperative Cognitive-Behavioral Patient Education Versus Standard Care for Lumbar Spinal Fusion Patients: Economic Evaluation Alongside a Randomized Controlled Trial," *Spine (Philadelphia, Pa. 1976),* vol. 41, no. 1, pp. 18-25, 2016, doi: 10.1097/BRS.0000000000001254.

[142] M. A. Erdogan *et al.*, "Patient-controlled Intermittent Epidural Bolus Versus Epidural Infusion for Posterior Spinal Fusion After Adolescent Idiopathic Scoliosis: Prospective, Randomized, Double-blinded Study," *Spine (Philadelphia, Pa. 1976),* vol. 42, no. 12, pp. 882-886, 2017, doi: 10.1097/BRS.0000000000001937.

[143] H.-J. M. D. Kim *et al.*, "Biomechanical advantages of robot-assisted pedicle screw fixation in posterior lumbar interbody fusion compared with freehand technique in a prospective randomized controlled trial—perspective for patient-specific finite element analysis," *The spine journal,* vol. 17, no. 5, pp. 671-680, 2016, doi: 10.1016/j.spinee.2016.11.010.

[144] D. M. Molinares *et al.*, "Is the lateral jack-knife position responsible for cases of transient neurapraxia?," (in eng), *J Neurosurg Spine,* vol. 24, no. 1, pp. 189-96, Jan 2016, doi: 10.3171/2015.3.Spine14928.

[145] E. K. Hoff, P. Strube, M. Pumberger, R. K. Zahn, and M. Putzier, "ALIF and total disc replacement versus 2-level circumferential fusion with TLIF: a prospective, randomized, clinical and radiological trial," *European spine journal,* vol. 25, no. 5, pp. 1558-1566, 2015, doi: 10.1007/s00586-015-3852-y.

[146] M. M. D. Putzier, T. M. D. Hartwig, E. K. M. D. Hoff, F. M. D. Streitparth, and P. M. D. Strube, "Minimally invasive TLIF leads to increased muscle sparing of the multifidus muscle but not the longissimus muscle compared with conventional PLIF—a prospective randomized clinical trial," *The spine journal,* vol. 16, no. 7, pp. 811-819, 2015, doi: 10.1016/j.spinee.2015.07.460.

[147] S. Kearing, S. Z. Berg, and J. D. Lurie, "Can Decision Support Help Patients With Spinal Stenosis Make a Treatment Choice?: A Prospective Study Assessing the Impact of a Patient Decision Aid and Health Coaching," *Spine (Philadelphia, Pa. 1976),* vol. 41, no. 7, pp. 563-567, 2016, doi: 10.1097/BRS.0000000000001272.

[148] D. Cankaya, M. Balci, A. Deveci, B. Yoldas, A. Tuncel, and Y. Tabak, "Better life quality and sexual function in men and their female partners with short-segment posterior fixation in the treatment of thoracolumbar junction burst fractures," *European spine journal,* vol. 25, no. 4, pp. 1128-1134, 2015, doi: 10.1007/s00586-015-4145-1.

[149] M. Perelló, D. Artés, C. Pascuets, E. Esteban, and A. M. Ey, "Prolonged Perioperative Low-Dose Ketamine Does Not Improve Short and Long-term Outcomes After Pediatric Idiopathic Scoliosis Surgery," *Spine (Philadelphia, Pa. 1976),* vol. 42, no. 5, pp. E304-E312, 2016, doi: 10.1097/BRS.0000000000001772.

[150] C. M. Bono *et al.*, "The effect of short (2-weeks) versus long (6-weeks) post-operative restrictions following lumbar discectomy: a prospective randomized control trial," *European spine journal,* vol. 26, no. 3, pp. 905-912, 2016, doi: 10.1007/s00586-016-4821-9.

[151] J. H. Lee *et al.*, "Comparison of fusion rate and clinical results between CaO-SiO2-P2O5-B2O3 bioactive glass ceramics spacer with titanium cages in posterior lumbar interbody fusion," *The spine journal,* vol. 16, no. 11, pp. 1367-1376, 2016, doi: 10.1016/j.spinee.2016.07.531.

[152] W. Wang *et al.*, "Tranexamic Acid Decreases Visible and Hidden Blood Loss Without Affecting Prethrombotic State Molecular Markers in Transforaminal Thoracic Interbody Fusion for Treatment of Thoracolumbar Fracture-Dislocation," *Spine (Philadelphia, Pa. 1976),* vol. 43, no. 13, pp. E734-E739, 2018, doi: 10.1097/BRS.0000000000002491.

[153] G. Nayar *et al.*, "Pedicle screw placement accuracy using ultra-low radiation imaging with image enhancement versus conventional fluoroscopy in minimally invasive transforaminal lumbar interbody fusion: an internally randomized controlled trial," (in English), *Journal of Neurosurgery: Spine SPI,* vol. 28, no. 2, pp. 186-193, 01 Feb. 2018 2018, doi: 10.3171/2017.5.Spine17123.

[154] H. Sarig Bahat, K. Croft, C. Carter, A. Hoddinott, E. Sprecher, and J. Treleaven, "Remote kinematic training for patients with chronic neck pain: a randomised controlled trial," *European spine journal,* vol. 27, no. 6, pp. 1309-1323, 2017, doi: 10.1007/s00586-017-5323-0.

[155] E.-Z. Yang *et al.*, "An RCT study comparing the clinical and radiological outcomes with the use of PLIF or TLIF after instrumented reduction in adult isthmic spondylolisthesis," *European spine journal,* vol. 25, no. 5, pp. 1587-1594, 2015, doi: 10.1007/s00586-015-4341-z.

[156] J. Greze *et al.*, "Does continuous wound infiltration enhance baseline intravenous multimodal analgesia after posterior spinal fusion surgery? A randomized, double-blinded, placebo-controlled study," *European spine journal,* vol. 26, no. 3, pp. 832-839, 2016, doi: 10.1007/s00586-016-4428-1.

[157] M. J. Desai *et al.*, "A Prospective, Randomized, Multicenter, Open-label Clinical Trial Comparing Intradiscal Biacuplasty to Conventional Medical Management for Discogenic Lumbar Back Pain," *Spine (Philadelphia, Pa. 1976),* vol. 41, no. 13, pp. 1065-1074, 2016, doi: 10.1097/BRS.0000000000001412.

[158] F. S. M. Araimo Morselli *et al.*, "Intrathecal Versus Intravenous Morphine in Minimally Invasive Posterior Lumbar Fusion: A Blinded Randomized Comparative Prospective Study," *Spine (Philadelphia, Pa. 1976),* vol. 42, no. 5, pp. 281-284, 2017, doi: 10.1097/BRS.0000000000001733.

[159] P. Strube, M. Putzier, F. Streitparth, E. K. Hoff, and T. Hartwig, "Postoperative posterior lumbar muscle changes and their relationship to segmental motion preservation or restriction: a randomized prospective study," (in eng), *J Neurosurg Spine,* vol. 24, no. 1, pp. 25-31, Jan 2016, doi: 10.3171/2015.3.Spine14997.

[160] S. Y. Park, H. S. An, S. H. Lee, S. W. Suh, J. L. Kim, and S. J. Yoon, "A prospective randomized comparative study of postoperative pain control using an epidural catheter in patients undergoing posterior lumbar interbody fusion," *European spine journal,* vol. 25, no. 5, pp. 1601-1607, 2016, doi: 10.1007/s00586-016-4385-8.

[161] J. Kesänen, H. Leino-Kilpi, T. Lund, L. Montin, P. Puukka, and K. Valkeapää, "Increased preoperative knowledge reduces surgery-related anxiety: a randomised clinical trial in 100 spinal stenosis patients," *European spine journal,* vol. 26, no. 10, pp. 2520-2528, 2017, doi: 10.1007/s00586-017-4963-4.

[162] N. H. vonderHoeh, A. Voelker, and C.-E. Heyde, "Results of lumbar spondylodeses using different bone grafting materials after transforaminal lumbar interbody fusion (TLIF)," *European spine journal,* vol. 26, no. 11, pp. 2835-2842, 2017, doi: 10.1007/s00586-017-5145-0.

[163] P.-I. Hung, M.-C. Chang, P.-H. Chou, H.-H. Lin, S.-T. Wang, and C.-L. Liu, "Is a drain tube necessary for minimally invasive lumbar spine fusion surgery?," *European spine journal,* vol. 26, no. 3, pp. 733-737, 2016, doi: 10.1007/s00586-016-4672-4.

[164] A. D. Norbye, A. V. Omdal, M. E. Nygaard, U. Romild, G. Eldøen, and R. Midgard, "Do Patients With Chronic Low Back Pain Benefit From Early Intervention Regarding Absence From Work?: A Randomized, Controlled, Single-Center Pilot Study," *Spine (Philadelphia, Pa. 1976),* vol. 41, no. 21, pp. E1257-E1264, 2016, doi: 10.1097/BRS.0000000000001878.

[165] Y. Matsuyama, K. Chiba, H. Iwata, T. Seo, and Y. Toyama, "A multicenter, randomized, double-blind, dose-finding study of condoliase in patients with lumbar disc herniation," (in eng), *J Neurosurg Spine,* vol. 28, no. 5, pp. 499-511, May 2018, doi: 10.3171/2017.7.Spine161327.

[166] K. Høy, K. Truong, T. Andersen, and C. Bünger, "Addition of TLIF does not improve outcome over standard posterior instrumented fusion. 5–10 years long-term Follow-up: results from a RCT," *European spine journal,* vol. 26, no. 3, pp. 658-665, 2016, doi: 10.1007/s00586-016-4592-3.

[167] E.-Z. Yang *et al.*, "Percutaneous Vertebroplasty Versus Conservative Treatment in Aged Patients With Acute Osteoporotic Vertebral Compression Fractures: A Prospective Randomized Controlled Clinical Study," *Spine (Philadelphia, Pa. 1976),* vol. 41, no. 8, pp. 653-660, 2016, doi: 10.1097/BRS.0000000000001298.
